# Allopurinol reduces the risk of myocardial infarction (MI) in the elderly: a study of Medicare claims

**DOI:** 10.1186/s13075-016-1111-1

**Published:** 2016-09-22

**Authors:** Jasvinder A. Singh, Shaohua Yu

**Affiliations:** 1 Medicine Service, Birmingham VA Medical Center, Birmingham, AL USA; 2 Department of Medicine at School of Medicine, and Division of Epidemiology at School of Public Health, University of Alabama at Birmingham (UAB), Faculty Office Tower 805B, 510 20th Street S, Birmingham, AL 35294 USA; 3 Department of Orthopedic Surgery, Mayo Clinic College of Medicine, Rochester, MN USA

**Keywords:** Allopurinol, Myocardial infarction, MI, Risk factor, Pharmacoepidemiology, Elderly, Predictor, Coronary artery disease, CAD

## Abstract

**Background:**

Previous observational studies that have examined the association of allopurinol with myocardial infarction (MI) have provided contradictory results. One study showed that allopurinol reduced the risk, while another study showed an increased risk with allopurinol. Therefore, our objective was to assess whether allopurinol use is associated with a reduction in the risk of MI in the elderly.

**Method:**

We used the 2006–2012 5 % random sample of Medicare beneficiaries to study the association of new allopurinol initiation and the risk of incident MI in a cohort study. Multivariable-adjusted Cox regression models adjusted for age, gender, race, and Charlson index, in addition to various cardio-protective medications (beta-blockers, angiotensin-converting enzyme (ACE) inhibitors, diuretics, statins). We calculated hazard ratios (HRs) with 95 % confidence intervals (CIs). Sensitivity analyses adjusted for coronary artery disease (CAD) risk factors, including hypertension, hyperlipidemia, diabetes, and smoking.

**Results:**

Of the 29,298 episodes of incident allopurinol use, 1544 were associated with incident MI (5.3 % episodes). Allopurinol use was associated with reduced hazards of MI, with a HR of 0.85 (95 % CI, 0.77 to 0.95). Compared to no allopurinol use, longer durations of allopurinol use were associated with a lower HR of MI: 1–180 days, 0.98 (95 % CI, 0.84 to 1.14); 181 days to 2 years, 0.83 (95 % CI, 0.72 to 0.95); and >2 years, 0.70 (95 % CI, 0.56 to 0.88). Other factors associated with a higher hazard of MI were: age 75 to <85 years and ≥85 years, male gender, higher Charlson index score, and the use of an ACE inhibitor. Adjustment for CAD risk factors confirmed these findings.

**Conclusion:**

Incident allopurinol use was associated with a reduction in the risk of incident MI in the elderly. Longer durations of allopurinol use reduced the risk of incident MI incrementally. Future studies should assess the underlying mechanisms for MI prevention and assess the risk-benefit ratio for allopurinol use.

**Electronic supplementary material:**

The online version of this article (doi:10.1186/s13075-016-1111-1) contains supplementary material, which is available to authorized users.

## Background

Coronary artery disease (CAD) is the most common cardiovascular disease [1]. CAD is the leading cause of disability [2] and mortality worldwide [3]. Myocardial infarction (MI) is a common, acute manifestation of CAD [4]. The case fatality rate of MI remains high, despite a reduction over time [4, 5]. Thus, MI constitutes a significant public health burden.

Allopurinol is commonly used for the treatment of hyperuricemia [6, 7]. Allopurinol is a structural isomer of hypoxanthine, and its active metabolite, oxypurinol, competes with hypoxanthine for the enzyme xanthine oxidase, and leads to the lowering of uric acid production. In addition to its urate-lowering effect, recent studies have suggested other mechanisms of action, some dependent and some independent of this action [8–15]. Debate continues whether allopurinol use reduces the risk of MI and the magnitude of this effect.

Two studies that examined the association of allopurinol with MI provided contradictory results. In a population-based case-control study, de Abajo et al. [16] reported that allopurinol was associated with a lower risk of MI with a hazard ratio (HR) of 0.52. This contradicts the finding of an increased HR of 1.25 for a cardiovascular event requiring hospitalization (including MI, stroke, hypertension, etc.) with allopurinol use in a population-based study of patients with gout by Kok et al. [17]. Compared to the positive study with only MI as an outcome [16], the study by Kok et al. included a more diverse (composite) outcome, used a prevalent rather than an incident user design, and was limited to patients with gout [17]. The study by de Abajo et al. was limited to non-fatal MI and used a case-control design, which are important study limitations [16]. Thus, both studies had important limitations that make the interpretation of study results difficult. The contradictory findings leave an average reader unclear about whether allopurinol use reduces the risk of MI or not. To our knowledge, it is also not known whether the MI risk reduction with allopurinol varies by certain patient characteristics, such as age, gender, and race.

Therefore, our objective was to assess whether allopurinol use was associated with a reduction in the risk of MI in the elderly. We hypothesized that (1) allopurinol use and (2) allopurinol use duration will each be independently associated with a reduction in the risk of MI. We also explored whether MI risk reduction with allopurinol varies by age, gender, and race.

## Methods

### Study cohort and population of interest

This retrospective cohort study used claims data from the 5 % random sample of Medicare beneficiaries from 2006 to 2012. Data were obtained from the Centers for Medicare and Medicaid Services (CMS) Chronic Condition Data Warehouse. The insurance claims for each beneficiary including inpatient, outpatient, skilled nursing facility, noninstitutional provider, home health, hospice, durable medical equipment services, and prescription drugs were extracted alongside beneficiary’s demographic information. Eligible subjects for the cohort study were: Medicare beneficiaries who were 65 years of age or older; lived in the US; were enrolled continuously in traditional Medicare fee-for-service and pharmacy coverage (Parts A, B, and D) and not enrolled in Medicare Advantage Plan, who had new treatment with allopurinol (defined in the section below). The Institutional Review Board at the University of Alabama at Birmingham approved the study.

### Exposure definition and covariates

We defined new allopurinol treatment as a new-filled allopurinol prescription, with no allopurinol prescription filled during a look-back baseline period of 365 days. Each day of observation within each episode was labeled as exposed or non-exposed based upon the days supply for allopurinol prescription in pharmacy records after the beginning of the episode. We allowed up to 30 days stock carry over. Patients were considered exposed for 30 days after the end of the days supply to capture the attributable events, after which a new continuous allopurinol exposure period started. We categorized allopurinol use duration as none, 1–180 days, 181 days to 2 years, and longer than 2 years. A patient could contribute multiple allopurinol treatment episodes during different time periods.

We obtained several covariates from the Medicare denominator file: age at the index date of each episode, gender, race/ethnicity, residence, and comorbidity scores in the baseline period for each allopurinol treatment episode, which were derived using Charlson-Romano comorbidity index score, a validated measure of medical comorbidity developed for claims data [18]. We also adjusted for the use of medications for cardiovascular diseases (beta-blockers, angiotensin-converting enzyme (ACE) inhibitors, statins, and diuretics).

### Outcome

The study outcome was incident MI, defined as the first incidence of MI during the study period after the initiation of a new allopurinol prescription, identified by the presence of International Classification of Diseases, ninth revision, common modification (ICD-9-CM) code, 410.x1. Patients had to have no MI in the baseline period of 365 days. The follow-up for each treatment episode began on the earliest allopurinol treatment initiation date during the study period and ended on the earliest of the first date of MI, the first date of losing full Medicare coverage, the date of death, or the end of the study (31 December 2012).

### Statistical analyses

We calculated summary statistics for the cohort, by occurrence versus non-occurrence of MI, and by allopurinol exposure versus not. We performed Cox proportional hazard regression models to assess the association of incident allopurinol exposure (yes/no) or the duration of allopurinol use and incident MI. Multivariable analysis adjusted for age, gender, race, Charlson-Romano comorbidity score, and the use of medications for cardiovascular diseases (beta-blockers, ACE inhibitors, statins, and diuretics). We accounted for correlated episodes (patients could possibly contribute more than one episode of new allopurinol use) using the Huber-White “Sandwich” variance estimator [19] and calculated robust standard errors for all estimates. HRs and 95 % confidence intervals (CIs) were calculated.

Sensitivity analyses were performed adjusting for: 1) individual CAD risk factors including hypertension, hyperlipidemia, diabetes, and smoking, instead of Charlson-Romano comorbidity index; (2) additionally adjusting the previous sensitivity analyses for CAD and peripheral vascular disease (PVD); and (3) additionally adjusting the previous sensitivity analyses for aspirin and colchicine use.

In multivariable-adjusted subgroup analyses by age, gender, and race, the main model was adjusted for all factors (age, gender, race, Charlson-Romano comorbidity score, use of cardiovascular medications, allopurinol use or duration) except the factor of interest for each subgroup analysis, respectively (age, gender, race).

## Results

### Characteristics of the patient population

**Fig. 1:**
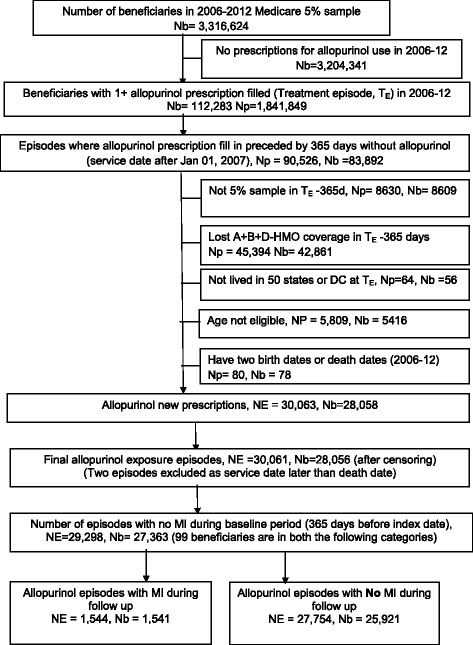
Flow-chart of study cohort of incident allopurinol users from 2006 to 2012 for a baseline of 365 days. *A + B + C-HMO*, *DC*, *MI* myocardial infarction, *Nb*, *NE* number of episodes, *Np*, *Number of prescriptions*, Nb, Number of beneficiaries

There were 29,298 episodes of incident allopurinol use, with no allopurinol use in the baseline period of 365 days (Table 1). Of these, 1544 allopurinol use episodes ended in an incident MI (5.3 % episodes), while the majority (94.7 %) did not (Fig. 1). Compared to incident allopurinol episodes that did not end in an MI, allopurinol episodes ending with an incident MI were associated with older age, a higher Charlson-Romano index (4.24 versus 2.98), male gender, and non-White race (Table 1). Table 2 shows MI incidence rate by allopurinol use and the duration of allopurinol use.

**Table 1:** Demographic and clinical characteristics of episodes of incident myocardial infarction* (MI) in allopurinol users

		Incident MI during follow-up		*P* value
	All observations	No	Yes	
Total episodes, *n*	**29,298**	**27,754**	**1544**	
Age (years), mean (SD)	76.6 (7.4)	**76.5 (7.4)**	**78.1 (7.6)**	**<0.0001**
Gender, *n* (%)				0.57
Male	14,502 (49.5)	13,727 (49.5)	775 (50.2)	
Female	14,796 (50.5)	14,027 (50.5)	769 (49.8)	
Race/ethnicity, *n* (%)				0.43
White	23,131 (79.0)	21,922 (79.0)	1209 (78.3)	
Black	3583 (12.2)	3,374 (12.2)	209 (13.5)	
Hispanic	613 (2.1)	578 (2.1)	35 (2.3)	
Asian	1,281 (4.4)	1,224 (4.4)	57 (3.7)	
Native American	100 (0.3)	93 (0.3)	7 (0.5)	
Other/unknown	590 (2.0)	563 (2.0)	27 (1.7)	
Region, *n* (%)				**<0.0001**
Northeast	4736 (16.2)	4428 (16.0)	308 (20.0)	
Midwest	7409 (25.3)	7003 (25.2)	406 (26.3)	
South	11,797 (40.3)	11,220 (40.4)	577 (37.4)	
West	5356 (18.3)	5103 (18.4)	253 (16.4)	
Charlson-Romano comorbidity Index Score, mean (SD)	3.65 (3.23)	**3.59 (3.21)**	**4.79 (3.41)**	**<0.0001**

*No baseline MI within 365 days before the index date of allopurinol episode

Significant differences and *p* values are in bold

*SD* standard deviation

**Table 2:** Incidence rate of myocardial infarction (MI) with incident allopurinol exposure* (yes versus no) and different allopurinol use duration periods

	Person-days of follow-up	MI cases	MI incidence rate per 1,000,000 person-days
Allopurinol use			
Yes	12,877,451	937	73
No	8,327,742	607	73
Allopurinol use duration			
0 days	8,327,742	607	73
1–180 days	5,902,851	514	87
181 days to 2 years	5,127,382	326	64
>2 years	1,847,218	97	53

*Baseline period was defined as 365 days during which patients could not have had an MI; baseline period for allopurinol was also 365 days before the index allopurinol prescription during which the patient could not have had a filled/refilled allopurinol prescription

Allopurinol use duration of “0 days” use represents the period where a person was not using allopurinol. This could be because they had not received their first prescription when we started observing them or because they went more than 30 days without getting a new prescription; they would begin contributing to the “0 days” category on day 31 of interruption of their allopurinol prescription

### Allopurinol use, allopurinol duration, and the risk of myocardial infarction

In patients with a new MI, those using allopurinol were older and had a higher Charlson comorbidity index compared to patients not using allopurinol (Table 3). Univariate associations are shown in Table 4. In multivariable-adjusted analyses, allopurinol use was associated with 15 % reduction in hazard of MI, with a HR of 0.85 (95 % CI, 0.77 to 0.95). Other factors associated with higher hazard of MI were: age 75 to <85 years and ≥85 years, male gender, higher Charlson index score, and the use of an ACE inhibitor (Table 4).

**Table 3:** Demographic characteristics and prevalence of comorbidities by allopurinol use in episodes with incident myocardial infarction (MI)

	No MI	MI		*P* value
		Not on allopurinol	On allopurinol	
Number of episodes	27,754	607	937	
Age (years), mean (SD)	76.5 (7.4)	**77.6 (7.5)**	**78.4 (7.6)**	**0.046**
Gender				0.13
Male	13,727 (49.5)	290 (47.8)	485 (51.8)	
Female	14,027 (50.5)	317 (52.2)	452 (48.2)	
Race				0.07
White	21,922 (79.0)	459 (75.6)	750 (80.0)	
Black	3374 (12.2)	97 (16.0)	112 (12.0)	
Others	2458 (8.8)	51 (8.4)	75 (8.0)	
Charlson-Romano comorbidity index score, mean (SD)	3.65 (3.23)	**4.52 (3.32)**	**4.96 (3.47)**	**0.01**
Comorbidity				
Diabetes	11076 (39.91)	290 (47.8)	482 (51.4)	0.16
Hypertension	23456 (84.5)	544 (89.6)	811 (89.6)	0.07
CVD	3442 (12.4)	106 (17.5)	184 (19.6)	0.28
PVD	4631 (16.7)	**133 (21.9)**	**247 (26.4)**	**0.047**

All numbers are *n* (%), unless specified otherwise

*P* value compares episodes with versus without allopurinol in patients who had an MI

Significant differences and *p* values are in bold

*CVD* cerebrovascular disease, *PVD* peripheral vascular disease, *SD* standard deviation

**Table 4:** Incident allopurinol use and the risk of incident myocardial infarction (MI)*

	Univariate		Multivariable-adjusted (model 1)**		Multivariable-adjusted (model 2)**	
	HR (95 % CI)	*P* value	HR (95 % CI)	*P* value	HR (95 % CI)	*P* value
Age (in years)						
65 to <75	Ref		Ref		Ref	
75 to <85	**1.45 (1.29,1.62)**	**<0.0001**	**1.36 (1.21, 1.52)**	**<0.0001**	**1.36 (1.21, 1.52)**	**<0.0001**
≥85	**2.08 (1.81, 2.38)**	**<0.0001**	**1.92 (1.67, 2.21)**	**<0.0001**	**1.92 (1.67, 2.21)**	**<0.0001**
Gender						
Male	Ref		Ref		Ref	
Female	0.96 (0.87, 1.06)	0.46	**0.87 (0.79, 0.96)**	**0.007**	**0.87 (0.79, 0.96)**	**0.007**
Race						
White	Ref		Ref		Ref	
Black	**1.18 (1.02, 1.37)**	**0.02**	1.14 (0.98, 1.32)	0.09	1.13 (0.97, 1.31)	0.11
Other	0.93 (0.78, 1.12)	0.45	0.93 (0.77, 1.11)	0.41	0.92 (0.77, 1.11)	0.37
Charlson- Romano index score	**1.15 (1.14, 1.17)**	**<0.0001**	**1.15 (1.13, 1.16)**	**<0.0001**	**1.15 (1.13, 1.16)**	**<0.0001**
Statins	0.86 (0.66, 1.11)	0.23	0.85 (0.66, 1.11)	0.23	0.85 (0.65, 1.11)	0.22
Beta blockers	1.05 (0.82, 1.33)	0.72	1.02 (0.79, 1.31)	0.90	1.01 (0.79, 1.31)	0.91
Diuretics	0.92 (0.73, 1.17)	0.51	0.84 (0.66, 1.08)	0.18	0.84 (0.66, 1.08)	0.17
ACE inhibitors	**1.33 (1.03, 1.70)**	**0.03**	**1.51 (1.17, 1.95)**	**0.002**	**1.51 (1.16, 1.95)**	**0.002**
Allopurinol use	**0.88 (0.79, 0.99)**	**0.02**	**0.85 (0.77, 0.95)**	**0.004**	-	-
Allopurinol use duration						
0 days	Ref		-	-	Ref	
1–180 days	1.00 (0.86, 1.16)	0.99	-	-	0.98 (0.84, 1.14)	0.76
181 days to 2 years	0.87 (0.76, 1.01)	0.06	-	-	**0.83 (0.72, 0.95)**	**0.009**
>2 years	**0.71 (0.56, 0.89)**	**0.003**	-	-	**0.70 (0.56, 0.88)**	**0.002**

*Incident MI is defined as no MI within the baseline period of 365 days before the index date of allopurinol episode

**Model 1 = allopurinol use (yes versus no) + age + race + gender + Charlson-Romano index score + beta blockers + diuretics + ACE inhibitors + statins

**Model 2 = allopurinol use duration + age + race + gender + Charlson-Romano index score + beta blockers + diuretics + ACE inhibitors + statins

Significant odds ratios and *p* values are in bold

Allopurinol use duration of “0 days” represents the period where a person was not using allopurinol. This could be because they had not received their first prescription when we started observing them or because they went more than 30 days without getting a new prescription; they would begin contributing to the “0 days” category on day 31 of interruption of their allopurinol prescription

- Not in the model, *ACE* angiotensin-converting enzyme, *CI* confidence interval, *HR* hazard ratio, *Ref* reference category

In a separate multivariable-adjusted model, compared to no allopurinol use, we found that longer allopurinol use duration was associated with a lower hazard of MI: 181 days to 2 years, 0.83 (95 % CI, 0.72 to 0.95) and >2 years, 0.70 (95 % CI, 0.56 to 0.88) (Table 4); allopurinol use for 1–180 days was not associated with reduction in hazard of MI.

Sensitivity analyses limited to patients with gout showed that the findings were unchanged with minimal/no attenuation of HRs (Table 5); 83 % of allopurinol users had a diagnosis of gout. Sensitivity analyses were performed adjusting for CAD risk factors, i.e., hypertension, hyperlipidemia, diabetes, and smoking (instead of Charlson-Romano index), and these confirmed the main findings: allopurinol use, 0.86 (0.77, 0.95), and allopurinol use duration (1–180 days, 0.97 (0.83, 1.13); 181 days to 2 years, 0.84 (0.73, 0.97); and >2 years, 0.69 (0.55, 0.87)) were significantly associated. Additional file 1 shows this in more detail. Sensitivity analyses that adjusted for PVD and CAD in addition to CAD risk factors confirmed the associations of allopurinol use and duration of allopurinol use with MI risk, with no further attenuation of HRs (see Additional file 2). Sensitivity analyses that further adjusted for aspirin and colchicine use showed minimal/no attenuation of HRs for allopurinol use and allopurinol use duration; neither aspirin, nor colchicine were significant in this model that adjusted for other covariates including CAD and PVD (see Additional file 3).

**Table 5:** Incident allopurinol use and the risk of incident myocardial infarction (MI)* limited to patients with a diagnosis of gout

	Univariate		Multivariable-adjusted (model 3)**		Multivariable-adjusted (model 4)**	
	HR (95 % CI)	*P* value	HR (95 % CI)	*P* value	HR (95 % CI)	*P* value
Age (in years)						
65 to <75	Ref		Ref		Ref	
75 to <85	**1.46 (1.29, 1.65)**	**<0.0001**	**1.37 (1.21, 1.55)**	**<0.0001**	**1.37 (1.21, 1.55)**	**<0.0001**
≥85	**2.04 (1.76, 2.36)**	**<0.0001**	**1.88 (1.62, 2.19)**	**<0.0001**	**1.88 (1.62, 2.19)**	**<0.0001**
Gender						
Male	Ref		Ref		Ref	
Female	0.98 (0.88, 1.10)	0.77	**0.88 (0.79, 0.99)**	**0.03**	**0.88 (0.79, 0.99)**	**0.03**
Race						
White	Ref		Ref		Ref	
Black	**1.22 (1.05, 1.43)**	**0.01**	1.16 (0.10, 1.36)	0.06	1.16 (0.99, 1.35)	0.07
Other	0.84 (0.70, 1.06)	0.16	0.86 (0.70, 1.05)	0.14	0.85 (0.69, 1.05)	0.12
Charlson- Romano index score	**1.15 (1.14, 1.1.7)**	**<0.0001**	**1.15 (1.13, 1.16)**	**<0.0001**	**1.15 (1.13, 1.16)**	**<0.0001**
Statins	0.87(0.65, 1.15)	0.31	0.84 (0.63, 1.13)	0.25	0.84 (0.63, 1.12)	0.24
Beta blockers	1.06 (0.81, 1.38)	0.68	1.01 (0.76, 1.34)	0.96	1.01 (0.76,1.34)	0.97
Diuretics	1.00 (0.77, 1.30)	0.99	0.91 (0.70, 1.20)	0.50	0.91 (0.69, 1.19)	0.49
ACE inhibitors	**1.43 (1.09, 1.88)**	**0.01**	**1.61 (1.22, 2.13)**	**0.0008**	**1.61 (1.22, 2.12)**	**0.009**
Allopurinol use	**0.84 (0.74, 0.94)**	**0.003**	**0.81 (0.72, 0.91)**	**0.0004**	-	-
Allopurinol use duration						
0 days	Ref		-	-	Ref	
1–180 days	0.94 (0.80, 1.11)	0.47			0.92 (0.78, 1.09)	0.33
181 days to 2 years	**0.83 (0.72, 0.97)**	**0.01**			**0.79 (0.68, 0.92)**	**0.002**
> 2 years	**0.68 (0.54, 0.86)**	**0.001**			**0.68 (0.53, 0.85)**	**0.001**

*Incident MI is defined as no MI within the baseline period of 365 days before the index date of allopurinol episode

**Model 3 = allopurinol use (yes versus no) + age + race + gender + Charlson-Romano index score + beta blockers + diuretics + ACE inhibitors + statins

**Model 4 = allopurinol use duration + age + race + gender + Charlson-Romano index score + beta blockers + diuretics + ACE inhibitors + statins

Significant odds ratios and *p* values are in bold

- Not in the model, *ACE* angiotensin-converting enzyme, *CI* confidence interval, *HR* hazard ratio, *Ref* reference category

Allopurinol use duration of “0 days” represents the period where a person was not using allopurinol. This could be because they had not received their first prescription when we started observing them or because they went more than 30 days without getting a new prescription; they would begin contributing to the “0 days” category on day 31 of interruption of their allopurinol prescription

### Subgroup analyses by age, gender, and race for risk reduction by allopurinol use duration

**Fig. 2:**
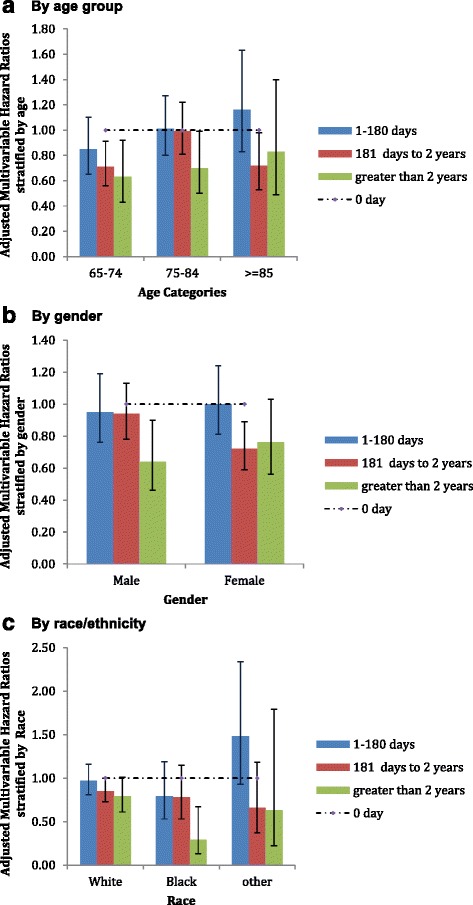
Multivariable-adjusted hazard ratios of MI by duration of allopurinol use by **a** age group, **b** gender and **c** race. For the multivariable-adjusted subgroup analyses by age, gender, and race, the main model was adjusted for all factors (age, gender, race, and Charlson-Romano comorbidity score) except the factor of interest, respectively, which was used to perform stratified analysis (age, gender, race)

In multivariable-adjusted subgroup analyses by age, gender, and race, allopurinol use durations of 181 days to 2 years and >2 years were associated with a reduction of hazard of MI, most evident for the age groups 65–74 and ≥85 years, male gender, and Blacks (Fig. 2).

## Discussion

In this study of Medicare claims we found that allopurinol use was independently associated with a lower risk of an incident MI. Compared to patients who did not use allopurinol, new allopurinol users had a 15 % lower hazard of an incident MI. We also found that a longer duration of allopurinol use was associated with a greater risk reduction in hazard for an incident MI, i.e., allopurinol use durations of 0.5–2 years and >2 years was associated with a 17 % and 30 % reduced hazard of an incident MI. Our study provides robust evidence of MI risk reduction with allopurinol use and suggests that it may have a cardioprotective action. Several study findings merit further discussion.

In previous studies, allopurinol use was associated with a 48 % reduction in the hazard (HR, 0.52) for an incident non-fatal MI using a Spanish database [16] versus a 25 % increase in the hazard (HR, 1.25) of a cardiovascular event requiring hospitalization (including MI, hypertension, stroke, etc.) in Taiwanese patients with gout [17]. The study showing an increased risk included a composite cardiovascular outcome (more diverse than MI) and used a prevalent user (rather than an incident user) design, both of which may have led to the lack of observation of a protective effect for MI [17]. On the other hand, the study by de Abajo et al. showing a protective allopurinol effect used a case-control study with matching (a less robust study design) and was limited to non-fatal MI, but used an incident user design [16]. The contradictory evidence from two population-based studies indicated that more evidence that is robust was needed, which will be crucial in deciding whether allopurinol use reduces the risk of MI or not. Other studies have shown a beneficial effect of allopurinol use (versus non-use) on mortality [20–22], heart failure readmission or death [23], and overall cardiovascular outcomes (including, but not limited to, MI) in patients with chronic kidney disease [24]; however, none specifically assessed MI only. A randomized, placebo-controlled, crossover trial included 65 adults with angiographically documented CAD, a positive exercise tolerance test, and stable chronic angina pectoris and randomized them to allopurinol (600 mg per day) or placebo for 6 weeks before crossover [13]. Allopurinol statistically significantly increased the median time to ST depression versus placebo (*p* = 0.0002; difference 43 s) and median total exercise time versus placebo (*p* = 0.0003) [13] showing its cardioprotective effect, providing one potential mechanism of the benefit we demonstrated. Similarly, a study comparing allopurinol to placebo in heart failure failed to show any benefit of allopurinol to placebo in patients with reduced ejection fraction  [25].

Our finding of a reduction in the risk of MI with allopurinol use in the elderly is based on a rigorous methodological approach using an incident user design. Use of a representative sample, adjustment for multiple covariates and potential confounders, and the robustness of estimates in sensitivity analyses, leads us to believe that our study findings are likely accurate and support a potential cardioprotective effect of allopurinol. To our knowledge, our study is amongst the first studies in the elderly population that show that allopurinol use is associated with a reduction in the risk of MI.

The mechanism of reduction in MI risk with allopurinol may be multi-fold. The anti-oxidant action of allopurinol [26–32] may be responsible for improving cardiac contractile function and preventing MI, similar to its beneficial effect on the progression of post-ischemic cardiomyopathy in mice [33]. Allopurinol improves endothelial function in renal failure, diabetes, sleep apnea, and heart failure [8–12, 34–43], also confirmed in a recent meta-analysis [44]. These mechanisms may delay the progression of atherosclerosis and/or prevent plaque instability [45]. Clinically, allopurinol has an anti-ischemic action in patients with stable, chronic angina [13], is associated with a reduction in left ventricular mass in patients with diabetes [14] and heart disease [15], and with a reduction in blood pressure [46, 47], which are potential mechanisms for its cardioprotective action. Other mechanisms of action for allopurinol include decreased macrophage interleukin 1-beta (IL1β) secretion upon the activation of NLRP3 inflammasome [48, 49], and the impairment in CD36-mediated TLR4/6-IRAK4/1 signaling [50], mechanisms that may contribute to cardiac risk [51].

Another important study finding was that the duration of allopurinol use was associated with a dose-dependent reduction in MI risk. In particular, compared to non-use, longer allopurinol use duration of >6 months to 2 years and >2 years were each associated with an independent reduction in the hazard of MI of 17 % and 30 %, respectively (HRs, 0.83 and 0.70). Surprisingly, allopurinol use <6 month was not associated with any reduction in the risk of MI, which might indicate a threshold of 6-month use for preventing MI. The reduction in hazard showed a response-gradient, which provides further support for the protective effect of allopurinol on incident MI, seen previously where long-term allopurinol use was associated with a lower hazard of cardiovascular events (including MI) at 0.43 (95 % CI, 0.21–0.88; *p* = 0.02) in an open-label extension of a randomized study [52]. Thus, longer term allopurinol use seems to be cardioprotective in patients with gout. Further support for a dose-response relationship for allopurinol use duration for cardioprotection is evident by observations of lower mortality risk in heart failure patients [22] and lower MI risk in the general population [16] with high-dose compared to low-dose allopurinol use. Whether the risk/benefit ratio of allopurinol becomes favorable due to potential cardioprotection in patients with asymptomatic hyperuricemia (without gout) remains to be seen.

The greatest reductions in the hazard of MI with allopurinol exposure were noted in those aged 65–74 and ≥85 years, those of female gender, and those of Black race, another interesting finding which needs confirmation and further study. A greater benefit in the elderly and a racial minority is particularly interesting, since these groups are usually at higher risk of worse MI outcomes [53, 54].

Our study has several strengths and limitations. Despite our efforts to control for various confounders, our study is subject to potential residual confounding due to the cohort study design. To avoid this bias, we controlled for multiple potential confounders and performed multiple sensitivity analyses that supported the robustness of our findings. Second, our database lacked information on other risk factors, such as over the counter aspirin use, body mass index and diet components, which can be viewed as risk factors for MI; however, risk reduction was confirmed in analyses adjusted for other traditional CAD risk factors including hypertension, hyperlipidemia, diabetes, and smoking, and in models that additionally adjusted for aspirin use (by prescription), and other cardiac and gout medications. Third, the number of MI events may have been too low to detect statistical significance of findings in certain subgroups, i.e., type II error; availability of the entire dataset would likely have overcome this limitation. However, use of a national 5 % Medicare sample is a standard approach in epidemiological research [55, 56], and we had in excess of 1500 events for the main analyses. Limited resources prevented us from analyses of 100 % Medicare data.

Fourth, the Medicare data do not allow us to investigate why allopurinol was prescribed and, although we know that 83 % of allopurinol users in our study had a diagnosis of gout, specific reasons for its use are not known and likely included other conditions such as hyperuricemia without gout, metabolic syndrome, renal stones, tumor lysis syndrome, etc. However, regardless of the reason for allopurinol prescription, our observation of its beneficial effect is generalizable to all US elderly who use allopurinol. We also showed with sensitivity analyses that these effects were similar in magnitude for elderly Americans with gout who used allopurinol. Finally, these data are derived from Medicare, and can only be generalized to the US elderly population, not the US general population. Fifty percent of the elderly using allopurinol are female, which is different to the usual institutional cohorts of allopurinol users dominated by men, since these studies include patients from all age groups; however, our sample is that of all US elderly aged 65 years and older.

Strengths of the study included the use of a representative US population, the ability to control for the common MI risk factors, robustness of findings in multiple sensitivity models, the use of an incident user design to avoid the biases of prevalent user design, and the examination of both the allopurinol exposure and the duration of allopurinol use.

## Conclusions

In conclusion, this study shows the cardioprotective effect of allopurinol in preventing MI in the elderly, and shows that this protective effect is evident after 6 months of allopurinol use and is more pronounced after 2 years of allopurinol use. The greatest reductions in the hazard of MI with allopurinol exposure were noted in age groups 65–74 and ≥85 years, females, and Black race. We used an incident user design and the associations noted were robust in multiple sensitivity analyses. These findings are generalizable to all US elderly people aged 65 years and older. Future studies need to examine the potential mechanisms of this cardioprotective effect of allopurinol use, which may uncover pathways either through urate reduction and/or other independent effects of allopurinol [57].

## Additional files

Additional file 1: Sensitivity analysis adjusted for CAD risk factors. Association of potential risk factors with the hazard of incident MI in patients who received allopurinol with no MI within 365 days before the index date of allopurinol use episode. This file shows that when the main model was additionally adjusted for CAD risk factors such as diabetes, hypertension, tobacco use disorder, and hyperlipidemia, instead of the Charlson-Romano index, the relationship of allopurinol use and allopurinol use duration with the risk of MI did not change. (DOCX 21 kb) Additional file 2: Sensitivity analysis adjusted for CAD risk factors, CVD and PVD. Association of various risk factors with the hazard of incident MI in patients who received allopurinol with no MI within 365 days before the index date of allopurinol episode. This file shows that when the main model was additionally adjusted for CAD risk factors (diabetes, hypertension, tobacco use disorder, and hyperlipidemia) instead of the Charlson-Romano index and CAD and peripheral vascular disease, the relationship of allopurinol use and allopurinol use duration with the risk of MI did not change. (DOCX 23 kb) Additional file 3: Sensitivity analysis adjusted for CAD risk factors, CVD, PVD, aspirin and colchicine use. Association of various risk factors with the hazard of incident MI in patients who received allopurinol with no MI within 365 days before the index date of allopurinol episode. This file shows that when the main model was additionally adjusted for CAD risk factors (diabetes, hypertension, tobacco use disorder, and hyperlipidemia) instead of the Charlson-Romano index, CAD and peripheral vascular disease, and aspirin and colchicine use, the relationship of allopurinol use and allopurinol use duration with the risk of MI did not change. (DOCX 23 kb)

## Abbreviations

ACE: Angiotensin-converting enzyme
CAD: Coronary artery disease
CI: confidence interval
HR: Hazard ratio
ICD-9-CM: International Classification of Diseases, ninth revision, common modification
MI: Myocardial infarction
PVD: Peripheral vascular disease
