# Supplementary material for: Allopurinol reduces the risk of myocardial infarction (MI) in the elderly: a study of Medicare claims
Source: Arthritis Res Ther. 2016 Sep 22;18:209. doi: 10.1186/s13075-016-1111-1 (PMC5032238; doi:10.1186/s13075-016-1111-1)
Supplement: Additional file 1: — Sensitivity analysis adjusted for CAD risk factors. Association of potential risk factors with the hazard of incident MI in patients who received allopurinol with no MI within 365 days before the index date of allopurinol use episode. This file shows that when the main model was additionally adjusted for CAD risk factors such as diabetes, hypertension, tobacco use disorder, and hyperlipidemia, instead of the Charlson-Romano index, the relationship of allopurinol use and allopurinol use duration with the risk of MI did not change. (DOCX 21 kb) [file 13075_2016_1111_MOESM1_ESM.docx]

**Additional File 1**. Sensitivity analysis adjusted for CAD risk factors: Association of potential risk factors with the hazard of incident MI in patients who received allopurinol with no MI within 365 days before the index date of allopurinol use episode

|  | Univariate | | Multivariable-adjusted (model 1*) | | Multivariable-adjusted (model 2*) | |
| --- | --- | --- | --- | --- | --- | --- |
|  | HR (95% CI) | P-value | HR (95% CI) | P-value | HR (95% CI) | P-value |
| Age |  |  |  |  |  |  |
| 65- <75 | Ref |  | Ref |  | Ref |  |
| 75- <85 | **1.45 (1.29,1.62)** | **<0.0001** | **1.50 (1.33, 1.68)** | **<0.0001** | **1.50 (1.34, 1.68)** | **<0.0001** |
| ≥85 | **2.08 (1.81, 2.38)** | **<0.0001** | **2.30 (2.00, 2.64)** | **<0.0001** | **2.30 (2.00, 2.65)** | **<0.0001** |
| Gender |  |  |  |  |  |  |
| Male | Ref |  | Ref |  | Ref |  |
| Female | 0.96 (0.87, 1.06) | 0.46 | 0.83 (0.75, 0.92) | 0.0003 | 0.83 (0.75, 0.92) | 0.0003 |
| Race |  |  |  |  |  |  |
| White | Ref |  | Ref |  | Ref |  |
| Black | **1.18 (1.02, 1.37)** | **0.02** | 1.16 (0.99, 1.34) | 0.06 | 1.15 (0.99, 1.34) | 0.07 |
| Other | 0.93 (0.78, 1.12) | 0.45 | **0.87 (0.73, 1.05)** | **0.15** | **0.87 (0.72, 1.04)** | **0.13** |
| Diabetes | **1.56 (1.41, 1.72)** | **<0.0001** | **1.62 (1.46, 1.79)** | **<0.0001** | **1.62 (1.46, 1.79)** | **<0.0001** |
| Hypertension | **1.32 (1.14, 1.54)** | **0.0003** | 1.19 (1.01, 1.40) | 0.03 | 1.19 (1.02, 1.40) | 0.03 |
| Hyperlipidemia | 1.01 (0.90, 1.13) | 0.87 | 0.94 (0.83, 1.06) | 0.30 | 0.94 (0.83, 1.06) | 0.29 |
| Tobacco use disorder | 1.13 (0.83, 1.55) | 0.44 | 1.26 (0.92, 1.72) | 0.16 | 1.26 (0.92, 1.72) | 0.16 |
| Beta blockers | 1.05 (0.82, 1.33) | 0.72 | 1.02 (0.79, 1.31) | 0.89 | 1.02 (0.79, 1.30) | 0.90 |
| Diuretics | 0.92 (0.73, 1.17) | 0.51 | 0.84 (0.66, 1.08) | 0.17 | 0.84 (0.66, 1.07) | 0.16 |
| ACE inhibitor | **1.33 (1.03, 1.70)** | **0.03** | **1.38 (1.06,1.79)** | **0.016** | **1.38 (1.06,1.78)** | **0.02** |
| Allopurinol | **0.88 (0.79, 0.99)** | **0.02** | **0.86 (0.77, 0.95)** | **0.005** | - | - |
| Allopurinol use duration |  |  |  |  |  |  |
| 0 days | Ref |  | - | - | Ref |  |
| 1-180 days | 1.00 (0.86, 1.16) | 0.99 | - | - | 0.97 (0.84, 1.14) | 0.73 |
| 181 days -2 years | 0.87 (0.76, 1.01) | 0.06 | - | - | **0.84 (0.73, 0.97)** | **0.02** |
| >2 years | **0.71 (0.56, 0.89)** | **0.003** | - | - | **0.69 (0.55, 0.87)** | **0.001** |

Model 1* is sensitivity analysis for Model 1 adjusted for Diabetes + hypertension+ Hyperlipidemia + tobacco use disorder instead of Charlson-Romano index score; in other words this model = Allopurinol use (yes/no) + age + race+ gender + Diabetes + hypertension+ Hyperlipidemia + tobacco use disorder + beta blockers + diuretics + ACE inhibitors

Model 2* is sensitivity analysis for Model 1 adjusted for Diabetes + hypertension+ Hyperlipidemia + tobacco use disorder instead of Charlson-Romano index score; in other words this model = Allopurinol use duration + age + race+ gender + Diabetes + hypertension + Hyperlipidemia + tobacco use disorder + beta blockers + diuretics + ACE inhibitors

**Significant odds ratios and p-values are in bold**
