# Supplementary material for: Allopurinol reduces the risk of myocardial infarction (MI) in the elderly: a study of Medicare claims
Source: Arthritis Res Ther. 2016 Sep 22;18:209. doi: 10.1186/s13075-016-1111-1 (PMC5032238; doi:10.1186/s13075-016-1111-1)
Supplement: Additional file 3: — Sensitivity analysis adjusted for CAD risk factors, CVD, PVD, aspirin and colchicine use. Association of various risk factors with the hazard of incident MI in patients who received allopurinol with no MI within 365 days before the index date of allopurinol episode. This file shows that when the main model was additionally adjusted for CAD risk factors (diabetes, hypertension, tobacco use disorder, and hyperlipidemia) instead of the Charlson-Romano index, CAD and peripheral vascular disease, and aspirin and colchicine use, the relationship of allopurinol use and allopurinol use duration with the risk of MI did not change. (DOCX 23 kb) [file 13075_2016_1111_MOESM3_ESM.docx]

**Additional File 3**. Sensitivity analysis adjusted for CAD risk factors, CVD, PVD, aspirin and colchicine use: Association of various risk factors with the hazard of incident MI in patients who received allopurinol with no MI within 365 days before the index date of allopurinol episode

|  | Univariate | | Multivariable-adjusted  (model 1***) | | Multivariable-adjusted  (model 2***) | |
| --- | --- | --- | --- | --- | --- | --- |
|  | HR (95% CI) | P-value | HR (95% CI) | P-value | HR (95% CI) | P-value |
| Age |  |  |  |  |  |  |
| 65- <75 | Ref |  | Ref |  | Ref |  |
| 75- <85 | **1.45 (1.29,1.62)** | **<0.0001** | **1.43 (1.27, 1.60)** | **<0.0001** | **1.43 (1.27, 1.60)** | **<0.0001** |
| ≥85 | **2.08 (1.81, 2.38)** | **<0.0001** | **2.12 (1.84, 2.45)** | **<0.0001** | **2.13 (1.85, 2.45)** | **<0.0001** |
| Gender |  |  |  |  |  |  |
| Male | Ref |  | Ref |  | Ref |  |
| Female | 0.96 (0.87, 1.06) | 0.46 | **0.84 (0.76, 0.94)** | **0.001** | **0.84 (0.76, 0.93)** | **0.001** |
| Race |  |  |  |  |  |  |
| White | Ref |  | Ref |  | Ref |  |
| Black | **1.18 (1.02, 1.37)** | **0.02** | 1.15 (0.99, 1.34) | 0.070 | 1.14 (0.98, 1.33) | 0.081 |
| Other | 0.93 (0.78, 1.12) | 0.45 | 0.89 (0.74, 1.07) | 0.21 | 0.88 (0.73, 1.10) | 0.18 |
| Diabetes | **1.56 (1.41, 1.72)** | **<0.0001** | **1.56 (1.41, 1.74)** | **<0.0001** | **1.57 (1.41, 1.74)** | **<0.0001** |
| Hypertension | **1.32 (1.14, 1.54)** | **0.0003** | 1.13 (0.97, 1.33) | 0.13 | 1.14 (0.97, 1.33) | 0.12 |
| PVD | **1.77 (1.58, 1.99)** | **<0.0001** | **1.49 (1.32, 1.68)** | **<0.0001** | 1.49 (1.32, 1.67) | **<0.0001** |
| CAD | **1.76 (1.55, 2.00)** | **<0.0001** | **1.49 (1.31, 1.70)** | **<0.0001** | 1.49 (1.31, 1.70) | **<0.0001** |
| Hyperlipidemia | 1.01 (0.90, 1.13) | 0.87 | 0.90 (0.80, 1.02) | 0.087 | 0.90 (0.80, 1.01) | 0.08 |
| Tobacco Disorder | 1.13 (0.83, 1.55) | 0.44 | 1.13 (0.82, 1.55) | 0.47 | 1.13 (0.82, 1.55) | 0.46 |
| Beta blockers | 1.05 (0.82, 1.33) | 0.72 | 1.01 (0.79, 1.30) | 0.94 | 1.01 (0.78, 1.29) | 0.96 |
| Diuretics | 0.92 (0.73, 1.17) | 0.51 | 0.84 (0.66, 1.08) | 0.18 | 0.84 (0.66, 1.08) | 0.17 |
| ACE inhibitor | **1.33 (1.03, 1.70)** | **0.03** | **1.39 (1.08, 1.81)** | **0.012** | **1.39 (1.07, 1.80)** | **0.013** |
| Aspirin | 0.86 (0.13, 6.00) | 0.88 | 0.67 (0.10, 4.76) | 0.69 | 0.67 (0.09, 4.73) | 0.69 |
| Colchicine | 1.15 (0.77, 1.72) | 0.50 | 1.15 (0.77, 1.72) | 0.50 | 1.14 (0.76, 1.71) | 0.52 |
| Allopurinol use | **0.88 (0.79, 0.99)** | **0.02** | **0.86 (0.77, 0.95)** | **0.005** | - | - |
| Allopurinol use duration |  |  |  |  |  |  |
| 0 days | Ref |  | - | - | Ref |  |
| 1-180 days | 1.00 (0.86, 1.16) | 0.99 | - | - | 0.97 (0.83, 1.13) | 0.72 |
| 181 days -2 years | 0.87 (0.76, 1.01) | 0.06 | - | - | **0.84 (0.73, 0.97)** | **0.014** |
| >2 years | **0.71 (0.56, 0.89)** | **0.003** | - | - | **0.69 (0.55, 0.87)** | **0.002** |

Model 1*** is sensitivity analysis for Model 1 adjusted for Diabetes + hypertension+ Hyperlipidemia + tobacco use disorder (instead of Charlson-Romano index score) + PVD + CAD + aspirin use + colchicine use; in other words this model = Allopurinol use (yes/no)+ age + race+ gender + Diabetes+ hypertension + PVD + CAD + Hyperlipidemia + tobacco use disorder + Aspirin + Colchicine + beta blockers + diuretics + ACE inhibitors

Model 2*** is sensitivity analysis for Model 1 adjusted for Diabetes + hypertension+ Hyperlipidemia + tobacco use disorder (instead of Charlson-Romano index score) + PVD + CAD + aspirin use + colchicine use; in other words this model = Allopurinol use duration + age + race+ gender + Diabetes + hypertension + PVD + CAD + Hyperlipidemia + tobacco use disorder + Aspirin + Colchicine + beta blockers + diuretics + ACE inhibitors

**Significant odds ratios and p-values are in bold**
